# Terpene Synthase Genes Originated from Bacteria through Horizontal Gene Transfer Contribute to Terpenoid Diversity in Fungi

**DOI:** 10.1038/s41598-019-45532-1

**Published:** 2019-06-25

**Authors:** Qidong Jia, Xinlu Chen, Tobias G. Köllner, Jan Rinkel, Jianyu Fu, Jessy Labbé, Wangdan Xiong, Jeroen S. Dickschat, Jonathan Gershenzon, Feng Chen

**Affiliations:** 1 Graduate School of Genome Science and Technology, University of Tennessee, Knoxville, TN 37996 USA; 2 Department of Plant Sciences, University of Tennessee, Knoxville, TN 37996 USA; 3 Max Planck Institute for Chemical Ecology, Hans-Knoell-Strasse 8, D-07745 Jena, Germany; 4 Kekulé-Institute of Organic Chemistry and Biochemistry, University of Bonn, Gerhard-Domagk-Straße 1, 53121 Bonn, Germany; 5 Tea Research Institute, Chinese Academy of Agricultural Sciences, Hangzhou, China; 6 Biosciences Division, Oak Ridge National Laboratory, Oak Ridge, TN 37831 USA; 7 Present Address: Center for Biotechnology, University of Nebraska-Lincoln, Lincoln, NE 68588 USA

**Keywords:** Enzymes, Natural products

## Abstract

Fungi are successful eukaryotes of wide distribution. They are known as rich producers of secondary metabolites, especially terpenoids, which are important for fungi-environment interactions. Horizontal gene transfer (HGT) is an important mechanism contributing to genetic innovation of fungi. However, it remains unclear whether HGT has played a role in creating the enormous chemical diversity of fungal terpenoids. Here we report that fungi have acquired terpene synthase genes (*TPSs*), which encode pivotal enzymes for terpenoid biosynthesis, from bacteria through HGT. Phylogenetic analysis placed the majority of fungal and bacterial *TPS* genes from diverse taxa into two clades, indicating ancient divergence. Nested in the bacterial *TPS* clade is a number of fungal *TPS* genes that are inferred as the outcome of HGT. These include a monophyletic clade of nine fungal *TPS* genes, designated as *BTPSL* for bacterial TPS-like genes, from eight species of related entomopathogenic fungi, including seven *TPSs* from six species in the genus *Metarhizium*. *In vitro* enzyme assays demonstrate that all seven *BTPSL* genes from the genus *Metarhizium* encode active enzymes with sesquiterpene synthase activities of two general product profiles. By analyzing the catalytic activity of two resurrected ancestral BTPSLs and one closely related bacterial TPS, the trajectory of functional evolution of BTPSLs after HGT from bacteria to fungi and functional divergence within *Metarhizium* could be traced. Using *M. brunneum* as a model species, both BTPSLs and typical fungal TPSs were demonstrated to be involved in the *in vivo* production of terpenoids, illustrating the general importance of HGT of *TPS* genes from bacteria as a mechanism contributing to terpenoid diversity in fungi.

## Introduction

Rich in taxonomic and phenotypic diversity, the fungal kingdom is one of the three large and dominant eukaryotic groups of the terrestrial ecosystems. Like animals and plants, fungi evolved as multicellular structures that concur to their adaptation to diverse ecological niches^1^. The origin and evolution in fungi reflect broader patterns of genome remodeling in a biochemical arms race with competitors and hosts, and thus their important ecological roles^2^. Consequently, one of the key characteristics of the fungal adaptation to various ecosystems is the broad diversity of secondary metabolites they produce^3^, including terpenoids. Fungal terpenoids have roles in diverse biological processes. For example, some are involved in various fungus-animal interactions; nematodes and arthropods being the main predator of fungi, they are the targets of terpenoid mycotoxins^4^. Some fungal terpenoids attract animals to facilitate spore dissemination^5^. Other fungal terpenoids have been demonstrated to mediate communication between fungi and bacteria^6^. Due to their biological activities, some fungal terpenoids are useful drugs^7^. Because of the chemical diversity, the diverse biological functions and the application of fungal terpenoids, it is of enormous interest to understand the mechanisms underlying terpenome biosynthesis in fungi^7^.

All terpenoids are synthesized from the same five-carbon precursors isopentenyl diphosphate (IPP) and dimethylallyl diphosphate (DMAPP). Three types of enzymes are known to be responsible for terpenoid diversification^8^. First, isoprenyl diphosphate synthases (IDS), which catalyzes the condensation of IPP and DMAPP to form isoprenyl diphosphates of various chain length, control the branching points of terpenoid biosynthesis. Second, terpene synthases (TPSs), which convert isoprenyl diphosphates to terpenes, are largely responsible for the diversity of terpene skeletons. Third, modifying enzymes, especially cytochrome P450s, add further structural diversity to terpenoids^9^. Besides these different types of enzymes, several mechanisms have been revealed to be important for terpenoid diversity. One mechanism is gene duplication and functional divergence, which is well supported by the presence of gene families of both TPS and P450s in fungi, plants and bacteria. Another mechanism is evolution of novel enzymes. This is best demonstrated by the identification of TPS-IDS hybrid type enzymes, which so far are known to occur only in fungi^10–15^.

In the general genetic innovation of fungi, besides gene duplication and functional divergence and creation of novel genes, another important mechanism is to acquire genes laterally, particularly from bacteria. Fungi and bacteria are often associated or interacting^16,17^. Previous studies (e.g.^18^) showed that the gene transfer between bacteria and eukaryotes can occur at a high magnitude, and HGT also plays an important role in eukaryotic adaptions and evolution^19^. However, whether HGT from bacteria has played a role in genetic innovation of terpenoids in fungi remains unknown. In principal, HGT of *TPS* genes could rapidly result in novel chemistry in the recipient organism. All organisms produce IPP, DMAPP and various types of isoprenyl diphosphates. As such, the substrates for TPSs are being produced by essentially all living organisms (also for the production of primary terpene metabolites). Therefore, *TPS* genes acquired through HGT can be functional in the recipient immediately after being acquired with its substrate readily available. If the acquisition of a *TPS* gene and consequently the ability of producing a suite of terpenoids provide fitness benefit for the recipient, such *TPS* genes of HGT would be retained and fixed in the population of the recipient. *TPS* genes are widely distributed in bacteria^20,21^. Based on this theoretical consideration, HGT of *TPS* genes from bacteria may have made important contribution to the vast diversity of terpenoids in fungi. This study is set to answer the question whether HGT of *TPS* genes from bacteria to fungi have occurred, and if so, to determine the contribution of such acquired *TPS* genes to terpenoid diversity in fungi.

## Results

### Phylogenetic analysis of fungal and bacterial TPSs and identification of cases of HGT events

**Figure 1:**
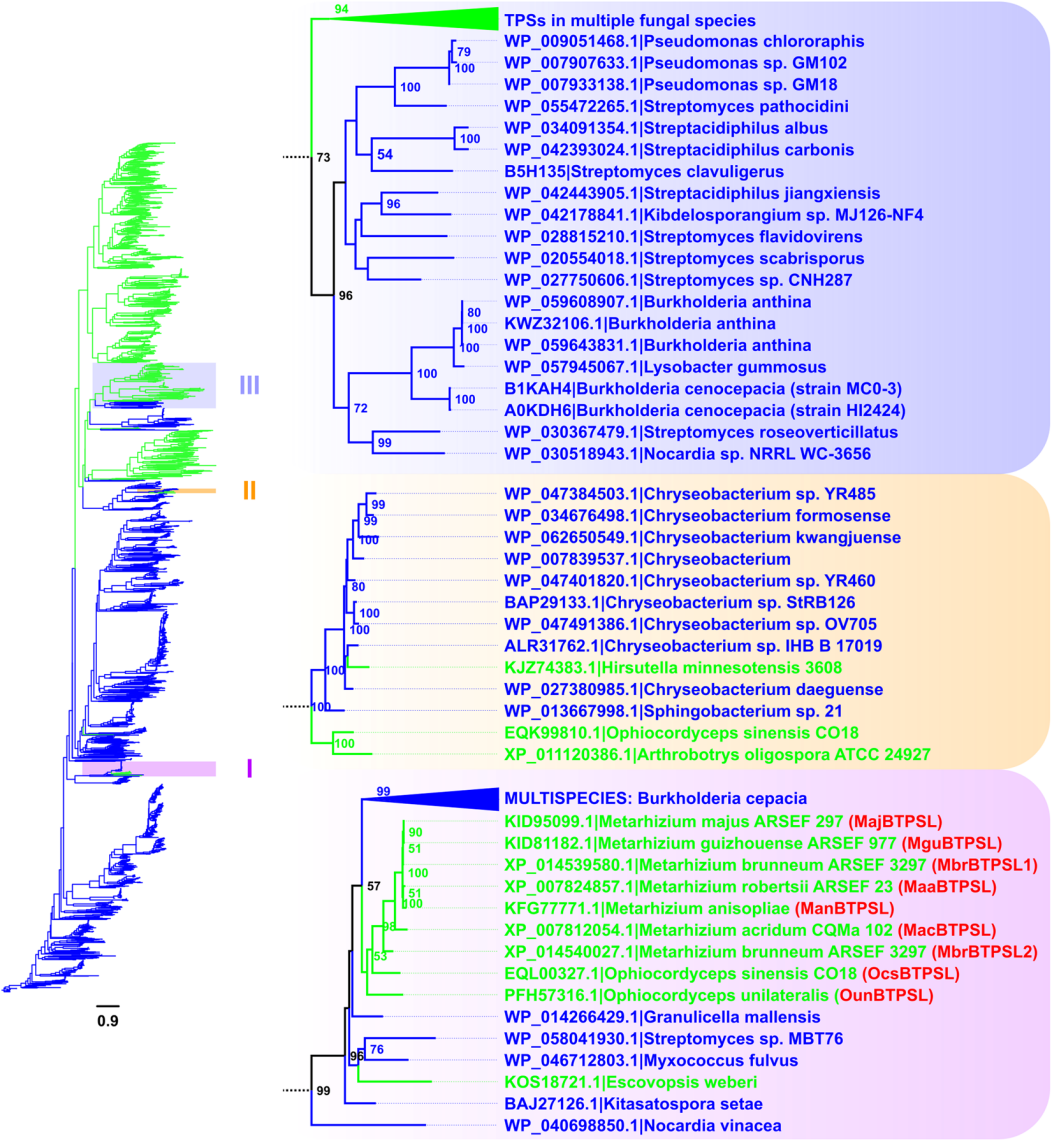
Maximum Likelihood phylogenetic tree of fungal and bacterial terpene synthases (TPSs). A total of 2443 TPSs from bacteria (Blue) and fungi (Green) was identified from the NCBI nr database. Three highlighted clades on the right are those with strong support in which fungal TPSs were embedded within bacterial TPSs (I and II) or vice versa (III). Bootstrap values are displayed next to the nodes, with values over 50% shown. Protein accession numbers and full species/strain names are shown as tips. Names of the 9 *BTPSL* genes in clade 1 are shown in red and in parentheses.

TPSs can be classified into different types based on reaction mechanism, substrate/product or domain structures^22^. This study focused on the main subclass of fungal and bacterial TPSs that contain well-defined Pfam domains of the microbial terpene synthase family: PF03936 (https://pfam.xfam.org/family/Terpene_synth_C) or PF06330 (https://pfam.xfam.org/family/TRI5). Using this criterion, an initial set of 348 fungal *TPS*s from 88 species and 287 bacterial *TPS*s from 140 species were obtained from the Pfam database (version 27.0)^23^. These sequences were then used as queries to search against the NCBI nr database using blastp. The final dataset includes 908 *TPS*s from fungi and 1535 *TPS*s from bacteria. To understand their evolutionary relatedness, fungal *TPS*s and bacterial *TPS*s were combined for phylogenetic reconstruction. According to the tree topology, the majority of fungal and bacterial *TPS*s clustered into two separate groups (Fig. 1). Nonetheless, a number of *TPS* genes were not placed within the phylogenetic branch representing their own kingdom. While some of the misplaced genes were poorly supported by the respective bootstrap values, three cases (I, II and III) were identified to have strong support (Fig. 1). They were inferred to be the outcome of HGT. We then focused on case I, which is a case of HGT of *TPS* gene from bacteria to fungi.

Within case I, nine fungal *TPS* genes form a monophyletic clade (Fig. 1). To distinguish these particular *TPS* genes from those typical fungal *TPS* genes, they were designated as bacterial terpene synthase-like (*BTPSL*) genes (Table S1). Seven of the nine *BTPSL* genes are from the genus *Metarhizium*, including *MacBTPSL* from *M. acridum*, *MajBTPSL* from *M. majus*, *MguBTPSL* from *M. guizhouense*, *ManBTPSL* from *M. anisopliae*, *MaaBTPSL* from *M. robertsii*, and two genes *MbrBTPSL1* and *MbrBTPSL2* from *M. brunneum*. The other two *BTPSL* genes are from the genus *Ophiocordyceps*, including *OcsBTPSL* from *O. sinensis* and *OunBTPSL* from *O. unilateralis*. It is notable that *Metarhizium* and *Ophiocordyceps* are two related genera, both are entomopathogenic^24^. All nine BTPSLs except OunBTPSL contain a highly conserved aspartate-rich “DDxxxD” motif located approximately 90 aa of their N-terminus and “NDxxSxxxE” motif at the C-terminus (Fig. S1). While *OcsBTPSL* and *OunBTPSL* each contains one intron, all seven *Metarhizium BTPSL* genes are intronless (Fig. S2).

### Evidence that *BTPSLs* are encoded by fungal nuclear genomes

**Figure 2:**
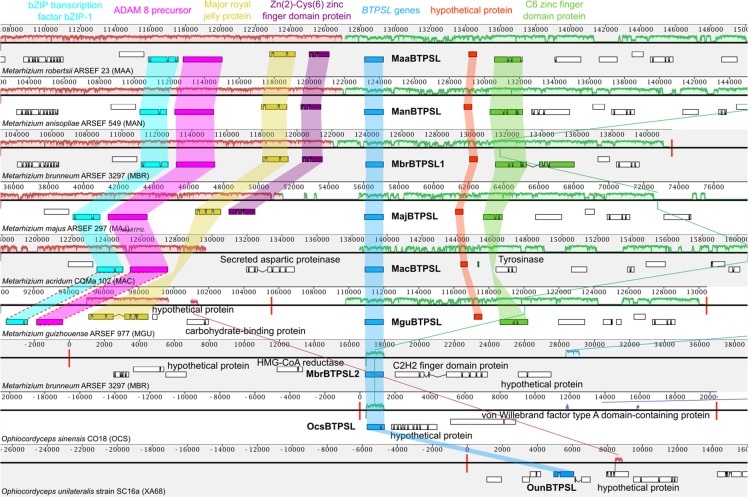
Synteny analysis of chromosomal regions containing *BTPSLs* from eight fungi species. Each horizontal profile indicates one genomic region with the height as the indicator of degree of sequence conservation. The white regions correspond to unaligned sequences. The regions that share the same color are locally collinear blocks. Below each profile are gene models and orthologous genes are colored and connected by shaded boxes in the same color. Red vertical lines delineate the ends of contigs. Genomes (*Metarhizium album*, *Metarhizium rileyi* and *Escovopsis weberi*) without regions similar to the region of *MaaBTPSL* were not aligned. Genes connected by the red dashed lines in the genome of *Metarhizium guizhouense* ARSEF 977 represent genes that are not visible in the current view.

To rule out the possibility of bacterial contamination for the presence of *BTPSL* genes in fungal genomes, two types of analysis were performed. The first analysis was to determine whether *BTPSL* and their neighbor genes from each species showed synteny. The scaffolds containing the 9 *BTPSLs* from 8 entomopathogenic fungi were aligned and each collinear set of matching regions was drawn as a contiguously colored local collinear block (LCB). The alignment shows that the surrounding regions of *BTPSLs* were collinear among all the *Metarhizium* species although some assembly was of low quality (Fig. 2). All *BTPSL*s were located in a single LCB with three exceptions, *MbrBTPSL2*, *OcsBTPSL* and *OunBTPSL*. Neighboring genes in the recipient genomes were displayed as rectangles. The gene order in the region surrounding *BTPSL* was found to be highly conserved across all members of the *Metarhizium* genus (Fig. 2). While one of the neighbor genes, major/yellow royal jelly protein, occurs in both eukaryotes and prokaryotes, all other flanking genes are non-bacterial.

**Figure 3:**
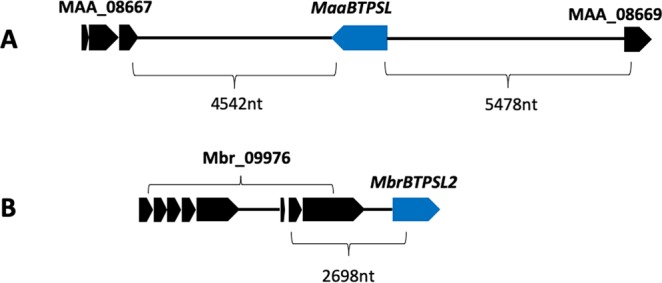
Verification that selected *BTPSL* genes are fungal nuclear genes. (**A**) Schematic genomic organization of *MaaBTPSL* with its neighboring genes MAA_08667 and MAA_08669 in *M. robertsii*. Two genomic regions spanning *BTPSL* gene and its neighboring genes were amplified using PCR from genome DNA and confirmed by sequencing. Number indicates distance in nucleotides (nt). (**B**) Schematic genomic organization of *MbrBTPSL2* with its neighboring gene Mbr_09976 in *M. brunneum*. A genomic region spanning *MbrBTPSL2* gene and Mbr_09976 was amplified using PCR from genome DNA and confirmed by sequencing. Number indicates distance in nucleotides (nt).

The other analysis was to amplify *BTPSL* genes from the fungal genome. *MaaBTPSL* and *MbrBTPSL2* were selected as two representatives to confirm their genomic locations. *MaaBTPSL* from *M. robertsii* was selected because it has orthologs in all other five species of *Metarhizium* (Fig. 3). Whereas *MbrBTPSL2* does not have an ortholog, it can be inferred to be derived from a gene duplication event that also leads to *MbrBTPSL1*. Genomic DNA sequences covering *MaaBTPSL* and *MbrBTPSL2* and their respective upstream and downstream genes as well as their intergenic regions were amplified using PCR. Amplified DNA fragments of *MaaBTPSL* (Fig. 3A) and *MbrBTPSL2* (Fig. 3B) were fully sequenced, and their positions were confirmed to be consistent with genome annotation.

### Catalytic activities of BTPSLs

**Figure 4:**
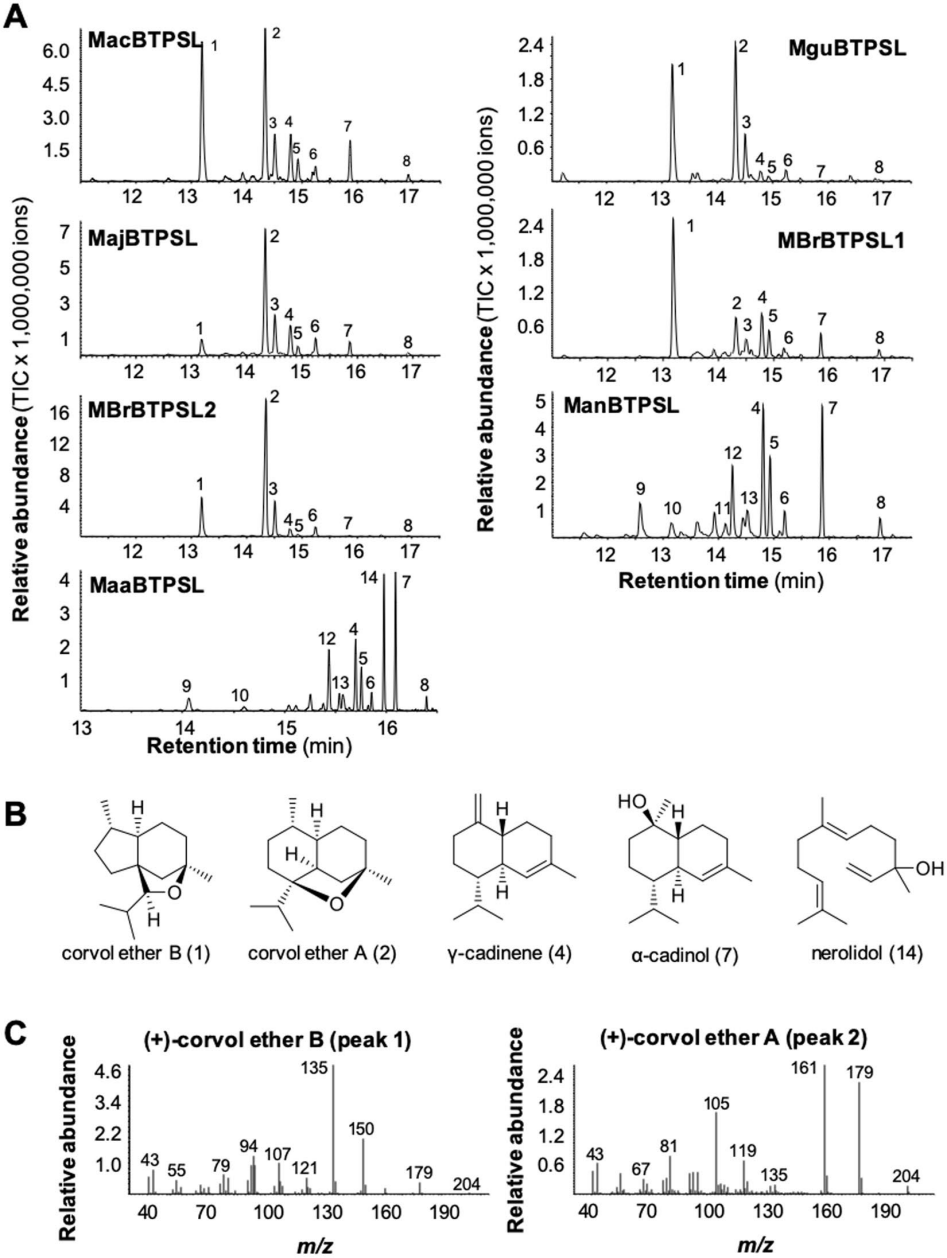
Biochemical characterization of BTPSL enzymes. (**A**) GC traces of sesquiterpene products of individual BTPSLs. 1, corvol ether B*; 2, corvol ether A*; 3, epizonarene; 4, γ-cadinene*; 5, δ-cadinene*; 6, α-cadinene*; 7, α-cadinol; 8, unidentified oxygen-containing sesquiterpene; 9, β-elemene*; 10, (*E*)-β-caryophyllene*; 11, γ-muurolene; 12, germacrene D; 13, α-muurolene*; 14, nerolidol. Compounds marked with asterisks (*) were identified using authentic standards. (**B**) Structures of the major sesquiterpene products of BTPSLs. Corvol ethers (**A** and **B**) are shown with absolute configuration, the other compounds are shown with relative configuration. (**C**) Mass spectra of (+)-corvol ether B (peak 1) and (+)-corvol ether A (peak 2).

With the verification that *BTPSL* genes are fungal nuclear genes, next, the seven *BTPSL* genes found in the six *Metarhizium* species were characterized for the catalytic activities of their encoded enzymes. Full-length coding sequences for each of the seven intron-less *BTPSL* genes were amplified from thei respecrtive genome DNA, cloned into a protein expression vector and heterologously expressed in *Escherichia coli*. Among the bacterial TPSs that are closed related to BTPSLs is one TPS BAJ27126 from the bacteria *Kitasatospora setae* (Fig. 1), which has been determined to be a sesquiterpene synthase^25^. As such, we chose to test recombinant BTPSs with (*E,E*)-farnesyl diphosphate (FPP), the substrate of sesquiterpene synthases. Each of the seven enzymes could convert FPP into a mixture of sesquiterpenes (Fig. 4A). Among the major products of these seven enzymes were corvol ether A and corvol ether B, γ-cadinene, α-cadinol and nerolidol (Fig. 4B) with the first two being unusual sesquiterpene ethers (Fig. 4C). Five of them, MacBTPSL, MguBTPSL, MajBTPSL, MbrBTPSL1, and MbrBTPSL2 produced similar mixtures consisting of eight sesquiterpenes that include corvol ether A, corvol ether B, epizonarene, γ-cadinene, δ-cadinene, α-cadinene, α-cadinol, and an unidentified sesquiterpene (Fig. 4A). While the product spectrum of MbrBTPSL1 was dominated by corvol ether B, MajBTPSL and MbrBTPSL2 produced mainly corvol ether A. MacBTPSL and MguBTPSL, however, formed both corvol ethers in similar concentrations. ManBTPSL and MaaBTPSL each produced 10 sesquiterpenes (Fig. 4A), including nine sesquiterpenes shared by the two enzymes: γ-cadinene, δ-cadinene, α-cadinene, α-cadinol, β-elemene, (*E*)-β-caryophyllene, germacrene D, and an unidentified oxygen-containing sesquiterpene. Besides, ManBTPSL produced γ-muurolene while MaaBTPSL produced nerolidol.

Interestingly, the sesquiterpene products of the bacterial TPS BAJ27126 from *K. setae* are also two corvol ethers^25^ (Fig. S3). The stereochemical course of the initial 1, 3-hydride shift in corvol ether biosynthesis was proved to be the same for the reaction catalyzed by MajBTPSL as for the enzyme from *K. setae*, which was investigated by conversion of (*R*)- and (*S*)-(1-^2^H)FPP (Fig. S4)^26^. These results point to the same absolute configuration of corvol ethers from both sources.

### Catalytic activity of resurrected ancestral *Metarhizium* MTPSLs and a TPS from a putative donor bacterium

**Figure 5:**
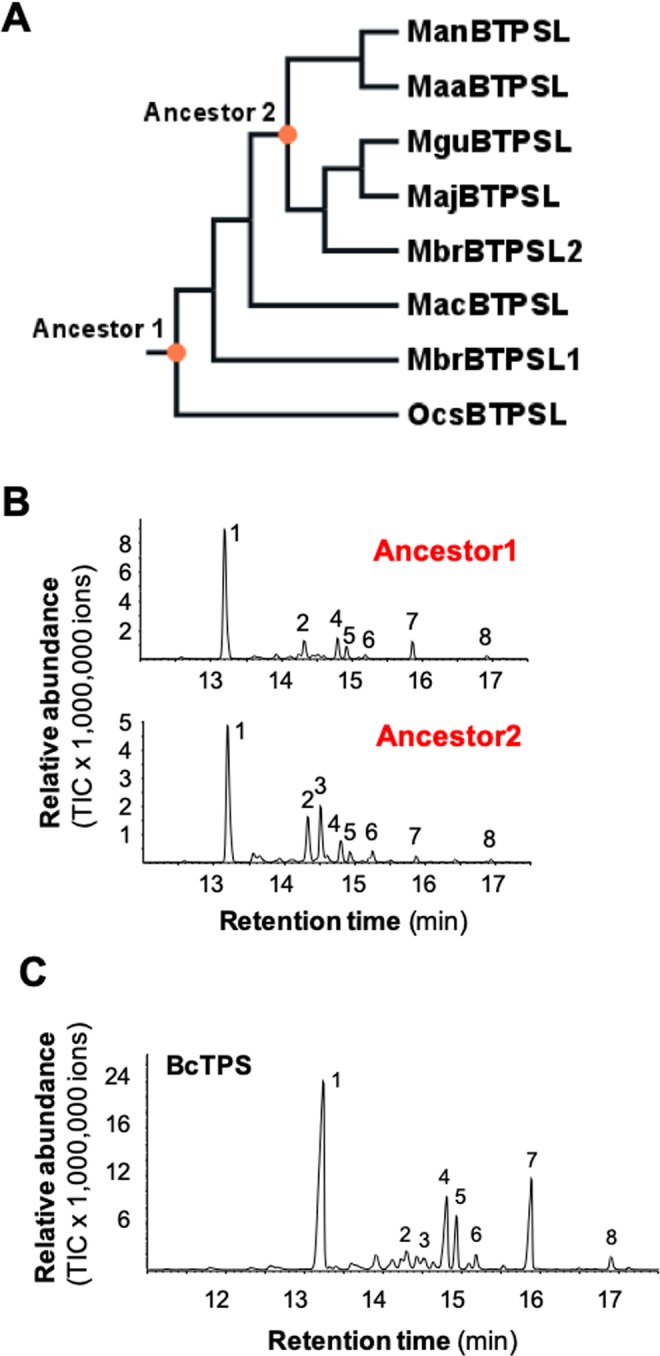
Catalytic activities of two resurrected ancestral BTPSLs and the TPS of a putative donor bacterium. (**A**) Phylogentic tree of seven BTPSLs from *Metarhizium* with OcsBTPSL from *Ophiocordyceps sinensis* as outgroup. Ancestor1 and Ancestor2 indicate the two ancestral BTPSLs. (**B**) GC chromatograms of assays products of Ancestor1 and Ancestor2 using farnesyl diphosphate as substrate. 1, corvol ether B*; 2, corvol ether A*; 3, epizonarene; 4, γ-cadinene*; 5, δ-cadinene*; 6, α-cadinene*; 7, α-cadinol; 8, unidentified oxygen-containing sesquiterpene. (**C**) Biochemical activity of terpene synthase (BcTPS) of the bacterium *Burkholderia capecia*. The gene was expressed in *Escherichia coli* and recombinant protein was incubated with the potential substrates (*E*,*E*)-farnesyl diphosphate (FPP). Enzyme products were analyzed using GC-MS. The total ion current (TIC) chromatograms are shown. 1, corvol ether B*; 2, corvol ether A*; 3, epizonarene; 4, γ-cadinene*; 5, δ-cadinene*; 7, α-cadinol; 8, unidentified oxygen-containing sesquiterpene. Compounds marked with asterisks (*) were identified using authentic standards.

By comparing their sesquiterpene products, the seven BTPSLs can be categorized into two groups. One group produced corvol ether B and/or corvol ether A as major product(s). This includes five enzymes. The other group, which includes ManBTPSL and MaaBTPSL, produced α-cadinol as a major product but do not produce corvol ether B and corvol ether A (Fig. 4A). This raised an intriguing question about the catalytic function of the ancestral protein of BTPSLs. To understand the trajectory of functional evolution of BTPSLs, we predicted the sequences of two ancestral genes. One gene, designated as *Ancestor 1*, is the putative ancestral gene in the common ancestor of *Metarhizium*. The other gene, designated as *Ancestor 2*, is a predicted ancestor gene before the divergence of two functional groups: *ManBTPSL/MaaBTPSL* and *MguBTPSL/MajBTPSL/MbrBTPSL2* (Fig. 5A). These two genes were synthesized and cloned into a protein expression vector and expressed in *E. coli*. Recombinant proteins of Ancestor1 and Ancestor2 were assayed with FPP and showed activity as BTPSLs. Both proteins produced corvol ether B, corvol ether A, γ-cadinene, δ-cadinene, α-cadinene, α-cadinol, and an unidentified oxygen-containing sesquiterpene (Fig. 5B). In addition, Ancestor2 formed another compound identified as epizonarene. Although corvol ether B was the most abundant compound in the product spectra of Ancestor1 and Ancestor2, Ancestor2 produced corvol ether A in relative higher concentrations than Ancestor 1.

Phylogenetic analysis showed that *BTPSL*s from the entomopathogenic fungi are most related to TPSs from the bacterial species in the genus *Burkholderia* (Fig. 1), suggesting an ancestor of *Burkholderia* or its related species might be the original donor of *BTPSL*s to entomopathogenic fungi. To understand the functional relatedness of *BTPSL*s and *Burkholderia TPS*s, we selected *Burkholderia cepacia* as a model species for experimental study. The *TPS* gene from *B. cepacia* was designated *BcTPS*. First, the full-length coding sequence of *BcTPS* was cloned from the genomic DNA of *B. cepacia* into a protein expression vector. *BcTPS* was then expressed in *E. coli* and its recombinant protein was assayed for terpene synthase activity using FPP as substrate. BcTPS had seven sesquiterpene products, including corvol ether B, corvol ether A, epizonarene, γ-cadinene, δ-cadinene, α-cadinol, and an unidentified oxygen-containing sesquiterpene (peak#8) (Fig. 5C).

### Both BTPSLs and typical fungal TPSs contribute to terpenoid diversity: a case study with *M. brunneum*

**Figure 6:**
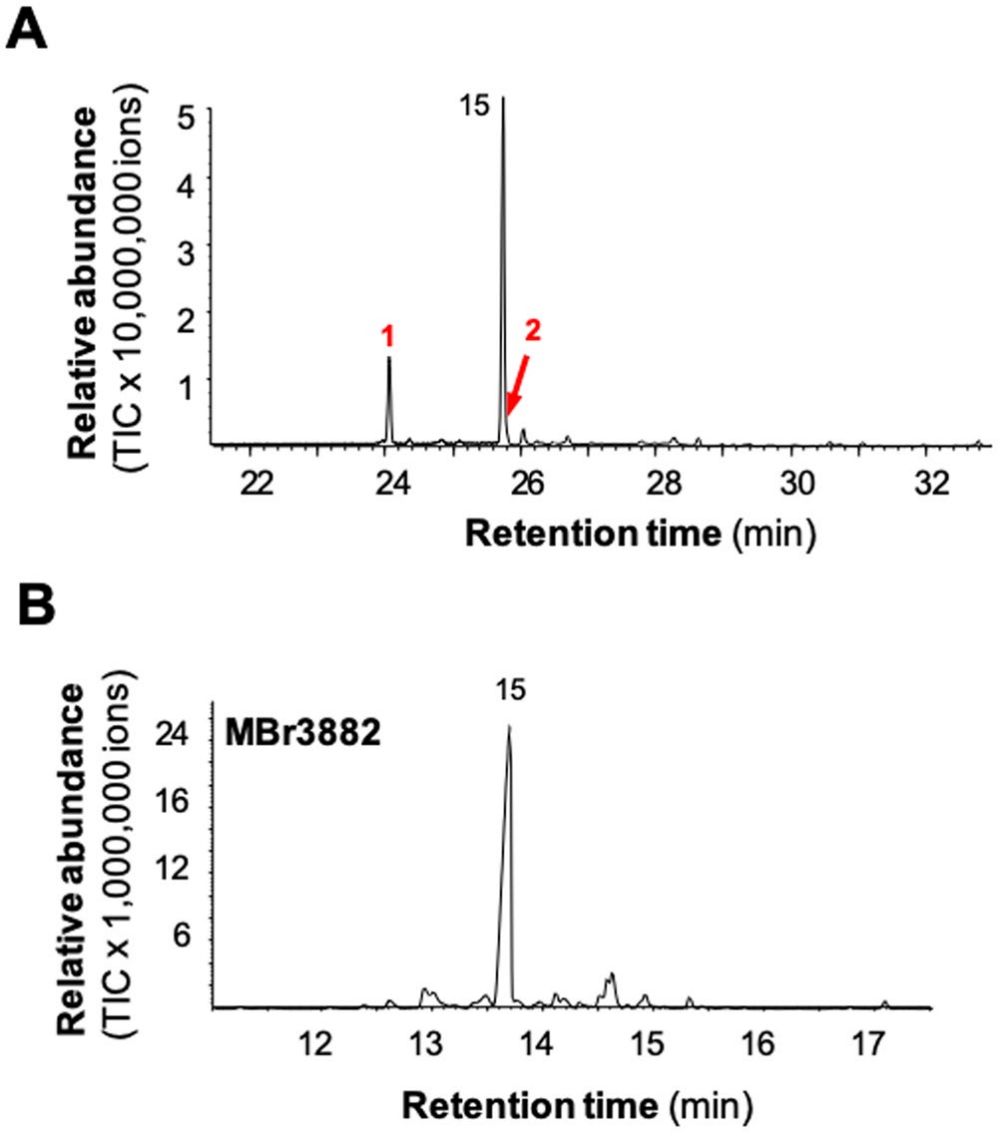
Volatile terpenes emitted from a liquid culture of *Metarhizium brunneum* and catalytic activity of typical terpene synthase from this species. (**A**) Three terpenes were detected from the liquid culture of *M. brunneum* using headspace volatile collection. 1, corvol ether B*; 2, corvol ether A*; 15, (*E*)-β-farnesene*. (**B**) Catalytic activity of typical fungal terpene synthase Mbr3882 from *M. brunneum*. 15, (*E*)-β-farnesene*. Compounds marked with asterisks (*) were identified using authentic standards.

Once *in vitro* functional characterization of BTPSLs was completed, next, we asked whether their catalytic activities are biologically relevant. To answer this question, *M. brunneum* was selected as a model species. Because the sesquiterpene products of BTPSLs are volatile compounds, we analyzed the headspace of *M. brunneum* culture in liquid medium. A number of sesquiterpenes were detected, including corvol ether B and corvol ether A, the products of MbrBTPS1 and MbrBTPSL2 (Fig. 6A). In addition, (*E*)-β-farnesene was detected as a major volatile. (*E*)-β-farnesene is not a product of MbrBTPSLs (Fig. 4), suggesting it is produced by other terpene synthases. The genome of *M. brunneum* contains two additional terpene synthase genes Mbr3882 and Mbr0969, which are clustered with the majority of fungal TPS (Fig. 1). They are termed typical fungal TPSs. To verify that (*E*)-β-farnesene is the product of typical fungal TPSs, the coding sequences of Mbr3882 and Mbr0969 were cloned into a protein expression vector and expressed in *E. coli*. Recombinant Mbr3882 and Mbr0969 were assayed for terpene synthase activity using FPP as substrate. While Mbr0968 was inactive with FPP, Mbr3882 catalyzed the formation of (*E*)-β-farnesene as major sesquiterpene product (Fig. 6B).

## Discussion

In this study, we report the identification of bacterial terpene synthase-like (*BTPSL*) genes in several entomopathogenic fungi originated from bacteria (Fig. 1) and the characterization of their functional evolution. These fungal *BTPSL* genes were verified to be fungal nuclear genes based on synteny analysis (Fig. 2) and genomic PCR analysis (Fig. 3). At the protein structural level, all BTPSLs possess an uncanonical aspartate-rich catalytic motif “DDxxxD” motif (Fig. S1), while in most typical fungal *TPS*s this motif is in the form of ‘DDxxD’, offering another indicator for their bacterial origin. The conservation in catalytic activities of most BTPSLs (Fig. 4A), their putative ancestor (Fig. 5B) and the related bacterial TPS (Fig. 5C) in producing the same rare sesquiterpenes corvol ether A and/or corvol ether B indicates the relatedness of these fungal and bacterial TPSs. Taken together, these results provide circumstantial evidence for HGT of *BTPSL* genes from bacteria to ancestral entomopathogenic fungi.

The monophyletic relationship of BTPSLs from six species of *Metarhizium* and two species of *Cordycepioideus* implies that the acquisition of *BTPLS* genes from bacteria may have occurred in the common ancestor of these two fungal lineages. Certainly, it is also possible that the genus *Metarhizium* and *Cordycepioideus* acquired their ancestral *BTPSL* genes independently, which is less parsimonious. Although the actual donor bacterium species is hard to identify, the ancestor of *Burkholderia* bacteria is the most likely source of this HGT based on the high sequence identity shared by *BTPSL* genes and *TPS*s in *Burkholderia* (Fig. S1). The genus *Burkholderia*, which belongs to the class β-proteobacteria and forms a monophyletic group, is widely distributed in the environment and often forms close associations with fungi^25^, providing a biological basis of the HGT of *TPS* genes.

Generally, the seven BTPSLs from *Metarhizium* can be categorized into two groups: one group producing corvol ether A and/or corvol ether B as the major product, the other group producing α-cadinol as a major product and corvol ether A/B as minor products. This suggests functional divergence of BTPSs within *Metarhizium*. Resurrecting the immediate ancestor (Ancestor2 in Fig. 5) of these two diverging groups and subsequent activity assays suggest that corvol ethers synthase is the ancestral activity (Fig. 5). This inference is further supported by the similarity between the catalytic activity of the resurrected common ancestor of *Metarhizium* BTPSLs, the catalytic activities of the majority of BTPSLs and that of the Ancestor1 (Fig. 5). The similarity between the catalytic activity of Ancestor 1 with that of the bacterial homolog of putative TPS donor (Fig. 5C) suggests that the ability to produce rare sesquiterpenes corvol ether A and/or corvol ether B by these related enzymes in fungi and bacteria has fitness benefit and probably has been under purifying selection.

By comparing the chemical profile of the *M. brunneum* culture with the product profiles of BTPSLs, it is evident that both BTPSLs and typical fungal TPSs contribute to the terpenoid diversity in these species. The majority of horizontally transferred genes get lost in the recipient genomes very quickly except those increasing the fitness of the recipient organism^27,28^. In *Metarhizium* species, the fact that these *BTPSL* genes are arranged in clusters along with other genes like transcription factors (a typical characteristic of fungal secondary metabolism) (Fig. 2) suggests that they may be under selection and their expression are probably fine-tuned by these transcription factors. While it can be generally hypothesized that the acquired *BTPSL* genes enhance the competitiveness and ecological specialization of the receipt fungal species, it will be interesting to test such hypothesis by elucidating the specific biological functions of *BTPSL* genes in respective fungal species.

Within case I, there is another fungal terpene synthase gene found from *Escovopsis weberi* (Fig. 1). Although the placement of this gene to this clade is highly supported, its position within this clade is not resolved (with a low support value of 31%). Additional evidence is needed to discern whether this particular *TPS* gene in *E. weberi* and the BTPSLs characterized in this study resulted from a single HGT event or two independent events. Besides the HGT case characterized in this study, there were two other strongly supported cases of *TPS* genes of incongruency in the phylogenetic analysis of fungal and bacterial TPSs (Fig. 1). One is another putative HGT of *TPS* genes from bacteria to fungi. Fungal *TPS*s in this case are from two nematode endoparasitic fungi (*Hirsutella minnesotensis* 3608 and *Arthrobotrys oligospora* ATCC 24927) and one caterpillar fungus *Ophiocordyceps sinensis* CO18. These three *TPS*s are clustered with many *TPS*s found in multiple Gram-negative bacteria species of the genus *Chryseobacterium* and one species of the genus *Sphingobacterium*. The other case of putative HGT of *TPS* genes is from fungi to bacteria. In this case, 20 bacterial *TPS*s clustering with fungal *TPS*s. Nearly half of them are from the family Streptomycetaceae, and others are from the genera of *Pseudomonas* and *Burkholderia*. It will be interesting to determine whether these two cases represent real case of HGT of *TPS* genes and if yes, to determine the biological importance of such HGT-derived *TPS* genes. In summary, this study not only showcases general importance of HGT of *TPS* genes from bacteria as a mechanism contributing to terpenoid diversity in fungi, but also opens the door to further understanding of HGT as a mechanism for contributing terpenoid diversity in diverse life forms.

## Materials and Methods

### Data sources and identification of terpene synthase genes

The Pfam database (version 27.0)^23^ was first searched by keyword “terpene synthase” and the protein sequences associated with the Pfam accessions “PF03936” (also known as “Terpene_synth_C”) and “PF06330” (also known as “TIR5”) were downloaded and the sequences from fungi and bacteria were extracted and complied. Next, each of the sequences was used as a query to search against the NCBI nr database using blastp at an e-value of 1e-5. The resulting dataset was then subjected to a HMMER search and only sequences containing PF03936 or PF06330 domain as the best-matched domain were kept for our further analysis.

### Multiple sequence alignments and phylogenetic inference

TPSs obtained from Pfam were first clustered at 100% sequence identity in each kingdom (bacteria and fungi) using CD-HIT^29^ to eliminate highly similar sequences within each kingdom. The corresponding C terminal domain sequences from bacterial and fungal TPSs were retrieved based on the coordinates predicted by HMMER. All multiple sequence alignments were made using MAFFT (v7.130b)^30^ in a highly accurate setting (L-INS-i) with 1000 iterations of improvement. The appropriate amino acid substitution model was determined using ProtTest version 3.4^31^ for each alignment according to Akaike information criterion (AIC) and Bayesian information criterion (BIC). The improved general amino acid substitution matrix with empirical base frequencies along with a gamma distribution (LG + G + F) was obtained as the most appropriate model for all protein datasets. Maximum likelihood analyses were performed using RAxML version 8.1.11^32^ with 1000 bootstrap replicates under the best substitution model for each dataset via the online CIPRES Science gateway portal^33^.

The maximum-likelihood tree shown in Fig. 4 was inferred from the codon alignment of *TPS* genes identified from several entomopathogenic fungi and *TPS* genes in bacteria that showing great similarity to *BTPSL*s. The codon alignment was generated using PAL2NAL^34^ from the MAFFT protein alignment (L-INS-I method with 1000 iterations of improvement) and the corresponding nucleotide sequences. The maximum-likelihood analysis was performed using PhyML 3.1^35^ with GTR + I + G nucleotide substitution model chosen by jModeltest2^36^ based on the AIC and BIC criteria. The robustness of the phylogenetic tree was estimated by bootstrapping with 1000 replicates.

For comparisons of sequences at the genome level, homologous contig sequences from each of seven species were aligned with sequence of MAA as the reference using the progressive alignment algorithm of the MAUVE Multiple Genome Aligner (version 2.4.0) at default settings^37^.

### Fungal cultures

*Metarhizium robertsii* (MAA) isolate (ARSEF 23), *M. brunneum* (MBR) isolate (ARSEF 3297), M. acridium (MAC) isolate (ARSEF 324), *M. majus* (MAJ) isolate (ARSEF 297), *M. guizhouense* (MGU) isolate (ARSEF 977) and *M*. *anisopliae* (MAN) isolate (ARSEF 549) were ordered from USDA ARS Collection of Entomopathogenic Fungal Cultures (ARSEF), Ithaca, New York. Lyophilized isolates were suspended with 500 µl sterilized distilled water and the final solution was cultured on Difco potato dextrose agar medium (PDA) at 28 °C for conidia development.

### Fungal *BTPSL* genes and bacterial *TPS* gene cloning

Mycelia of six fungal species developing from lyophilized isolates were used to isolate genomic DNA. A bacterial species homological to *Metarhizium*, *Burkholderia cepacia* (ATCC 25416) was ordered from the American Type Culture Collection (ATCC). The genomic DNA was extracted using GeneJET Plant Genomic DNA Purification Kit (https://www.thermofisher.com) according to the protocol recommended by the manufacturer. Primers were designed to amply the entire coding sequences of all seven *Metarhizium BTPSL* genes and one *B. cepacia TPS* gene (*BcTPS*) (Table S2). PCR products for fungal *BTPSL* genes and *BcTPS* gene were cloned into pEXP5-CT/TOPO vector (www.lifetechnologies.com) according to the protocol provided by the manufacturer and fully sequenced.

### Confirmation of *MbrBTPSL2* and *MaaBTPSL* with neighboring genes

Two *BTPSL* genes, *MbrBTPSL2* and *MaaBTPSL*, were selected to confirm their locations. For neighboring genes of *MbrBTPSL2*, forward primer MbrBTPSL2NBF was designed at the sixth intron area of MBR_09976, and reverse primer MbrBTPSL2NBR was selected around 340nt from the start codon of *MBrBTPSL*. For *MaaBTPSL*, two pairs of primers were designed to amplify partial MaaBTPSL and its downstream or upstream neighboring gene (Table S2). Genomic DNAs of *M. brunneum* and *M. robertsii* were used as template for PCR using PfuUltra II Fusion HS DNA polymerase (http://www.genomics.agilent.com). The DNA fragment was excised from agarose gel, purified, and cloned into pGEM-T EASY vector (https://www.promega.com) for sequencing.

### Biochemical characterization of BTPSLs from *Metarhizium* and BcTPS

The *E. coli* BL21 codon plus strain (http://www.lifetechnologies.com), transformed with the plasmid containing the open reading frame of *MacBTPSL*, *MajBTPSL*, *MguBTPSL*, *MbrBTPSL1*, *MbrBTPSL2*, *ManBTPSL*, *MaaBTPSL*, or *BcTPS,* was used for protein expression. The BL21 culture was grown in liquid LB at 37 °C until the culture reached 0.6 at OD_600_. Protein expression was induced by the addition of isopropylthio-β-galactoside (IPTG) to a final concentration of 1 mM. After 20 hours of incubation at 18 °C, cells were harvested by centrifugation at 6000 g, resuspended in protein extraction buffer (50 mM Mopso (pH7.0), 5 mM MgCl_2_, 5 mM Sodium ascorbate, 5 mM dithiothreitol, 0.5 mM PMSF and 10% (v/v) glycerol) and disrupted with a sonicator (Microson XL 2000; Misonix, Farmingdale, New York). Cell debris was removed by centrifugation at 13,000 rpm (30 min, 4 °C) and the supernatant was desalted by passage through a PD-10 Desalting Column (http://www.gelifesciences.com) into assay buffer (10 mM Mopso, pH7.0, 1 mM dithiothreitol, 10% (v/v) glycerol).

To determine the catalytic activity of fungal BTPSLs and BcTPS, enzyme assays were conducted in a Teflon-sealed, screw-capped 1 ml GC glass vial containing 40 µl of the bacterial extract and 60 µl assay buffer containing 10 µM (*E*,*E*)-FPP, 10 mM MgCl_2_, 0.2 mM NaWO_4_, and 0.1 mM NaF. A SPME (solid phase microextraction) fiber consisting of 100 µm polydimethylsiloxane (http://www.sigmaaldrich.com) was placed into the headspace of the vial for 45 min incubation at 30 °C to adsorb the TPS reaction products. Product analysis was conducted using an Agilent 6890 Series gas chromatograph (GC) coupled to an Agilent 5973 quadrupole mass selective detector (interface temp, 250 °C; quadrupole temp, 150 °C; source temp, 230 °C; electron energy, 70 eV). The GC was operated with a DB-5MS column (Agilent, Santa Clara, USA, 30 m × 0.25 mm × 0.25 µm). The sample (SPME) was directly injected without split at an initial oven temperature of 80 °C. The temperature was held for 3 min, then increased to 240 °C with a gradient of 7 °C min^−1^, and further increased to 300 °C with a gradient of 60 °C min^−1^ and a hold of 2 min. Compounds were identified by comparisons of retention times and mass spectra to those of authentic standards obtained from Fluka (Seelze, Germany), Roth (Karlsruhe, Germany), Sigma (St. Louis, MO, USA), or by reference spectra in the Wiley and National Institute of Standards and Technology libraries.

### *In vitro* identification of MajBTPSL products as corvol ethers

The gene for MajBTPSL was cloned into the expression vector pYE-express^38^ using homology arms (underlined) containing primers (GGCAGCCATATGGCTAG and CATGACTGGTGGAATGGAAAAACAAAGATTGAAAGCCCAACTTTC and CTCAGT and GGTGGTGGTGGTGGTGCTCGAGTCTAGACCAAGCTGCTCGTTGACTC) for homologous recombination in yeast^39^. The obtained plasmid was shuttled to *E. coli* BL21(DE3) and the successful insertion of the target gene was checked by sequencing. The transformant was grown in LB medium (10 g tryptone, 5 g yeast extract, 5 g NaCl, pH 7.2, 1 L H_2_O) supplied with kanamycin (50 mg/L) at 37 °C overnight. This preculture (1/100) was used to inoculate a LB-kanamycin main culture (200 mL), which was grown at 37 °C with shaking until OD_600_ = 0.4–0.6 was reached. The culture was cooled to 18 °C, before IPTG (0.4 mm final concentration) was added to induce expression of the recombinant protein. Shaking was continued at the same temperature overnight, before the cells were harvested by centrifugation (5000 × g, 4 °C, 10 min) and resuspended in binding buffer (5 mL; 20 mm Na_2_HPO_4_, 500 mm NaCl, 20 mm imidazole, 1 mm MgCl_2_, pH = 7.4, 4 °C). The cells were lysed by ultrasound (50% power, 4 °C, 5 × 30 s) and the cell debris was removed by centrifugation (5400 × g, 4 °C, 7 min). The soluble fraction was filtered and loaded on a Ni^2+^-NTA affinity column (Ni-NTA superflow, Qiagen, Venlo, Netherlands), which was washed with binding buffer (2 × 2 mL). The target protein was obtained by adding elution buffer to the column (2 × 1 mL; 20 mm Na_2_HPO_4_, 500 mm NaCl, 500 mm imidazole, 1 mm MgCl_2_, pH = 7.4, 4 °C). The obtained protein solution was directly used for incubation experiments. The substrates FPP, (1 *R*)-(1-^2^H)FPP and (1 *S*)-(1-^2^H)FPP^26^ (1 mg each) were dissolved in substrate buffer (1 mL; 25 mm NH_4_HCO_3_ in H_2_O) and diluted with binding buffer (2 mL) and incubation buffer (4 mL; 50 mm Tris/HCl, 10 mm MgCl_2_, 20% glycerol, pH = 8.2). The reaction was started by adding MajBTPSL elution fraction (1 mL). The samples were incubated for 3 h at 28 °C, before they were extracted using hexane. The organic phase was dried with MgSO_4_ and analyzed by GC-MS.

### Prediction of ancestral *TPS* genes

Ancestral sequence reconstruction was performed using FastML^40^. FastML requires the multiple sequence alignment and a related phylogenetic tree as input for predicting ancestral gene sequences. Multiple sequence alignments of *BTPSL* genes were performed using MAFFT and maximum likelihood phylogenetic trees prepared using and RAxML.

### Volatile profiling of *M. brunneum*

Conidia of *M. brunneum* developing from potato dextrose agar plate were transferred into a one-liter flask containing 200 ml of liquid potato dextrose broth. The fungal culture was grown at room temperature on a rotary shaker (100 rpm) for one week. A solid phase microextraction (SPME) fiber consisting of 100 µm polydimethylsiloxane (https://www.sigmaaldrich.com) was inserted into the headspace of the flask. The SPME fiber was directly inserted into the injector port of GC-MS after 16 h of collection. This analysis was repeated for three times.

## Supplementary information

supplemental data
