# Supplementary material for: Terpene Synthase Genes Originated from Bacteria through Horizontal Gene Transfer Contribute to Terpenoid Diversity in Fungi
Source: Sci Rep. 2019 Jun 25;9:9223. doi: 10.1038/s41598-019-45532-1 (PMC6592883; doi:10.1038/s41598-019-45532-1)
Supplement: Supplementary file 1 — supplemental data [file 41598_2019_45532_MOESM1_ESM.pdf]

Supplementary Information

Terpene Synthase Genes Originated from Bacteria through Horizontal Gene Transfer Contribute to Terpene Diversity in Fungi

Qidong Jia, Xinlu Chen, Tobias G. Köllner, Jan Rinkel, Jianyu Fu, Jessy Labbé, Wangdan Xiong, Jeroen S. Dickschat, Jonathan Gershenzon, and Feng Chen

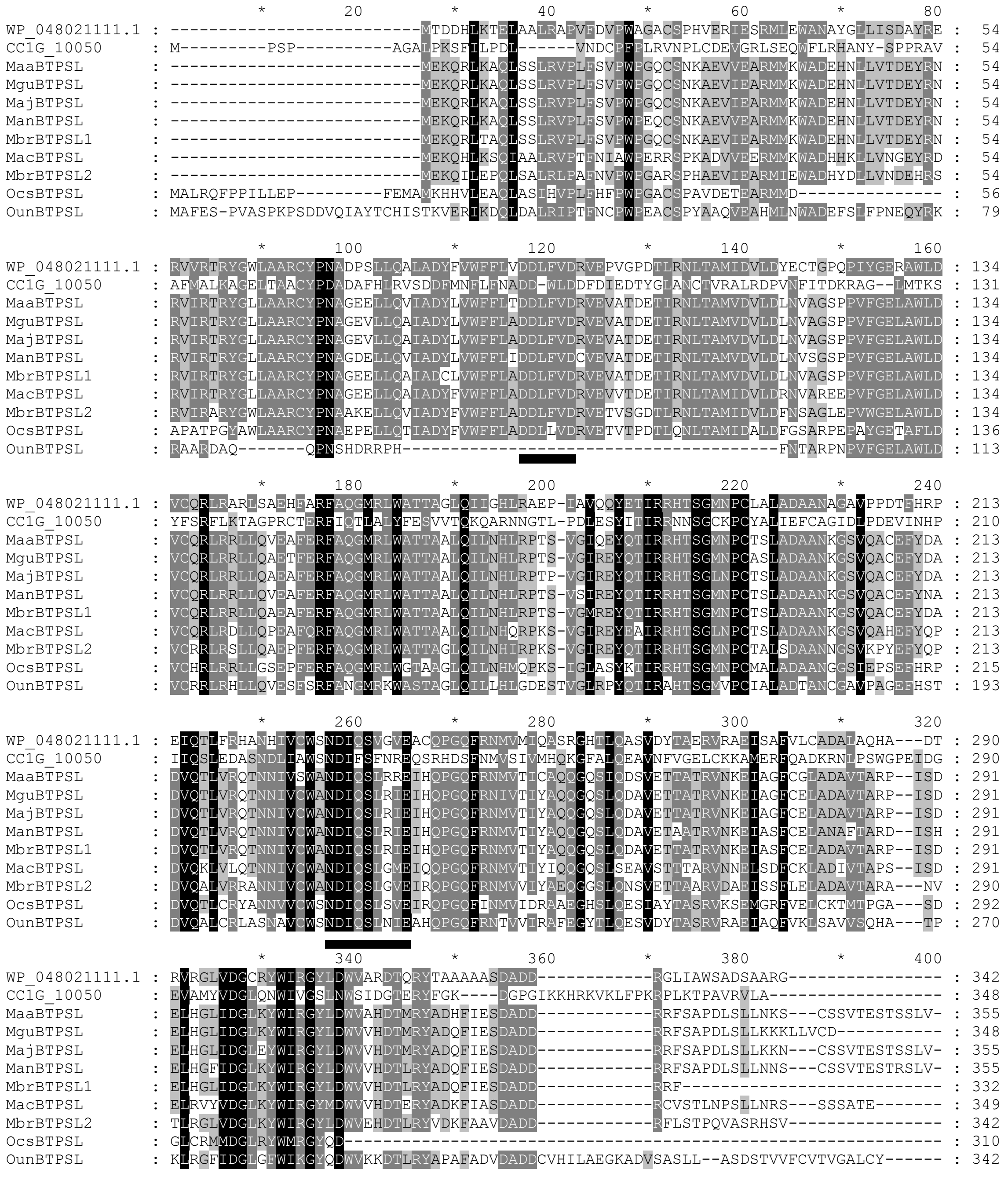

Fig S1. Multiple sequence alignment of selected terpene synthases. These includes 9 bacterial terpene synthase-like (BTPSL) proteins and representative terpene synthase WP_048021111.1 and CC1G_10050 from bacteria (Burkholderia) and fungi (*Coprinopsis cinerea* okayama7#130), respectively. The positions of DDxx(x)D and NDxxSxxxE motifs are indicated by black bars.

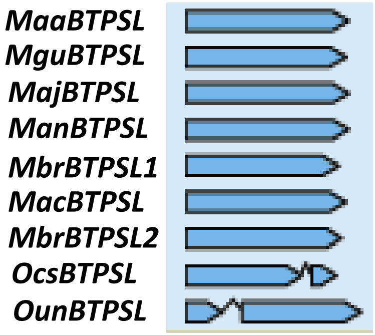

Fig S2. Intron-exon organization of bacterial terpene synthase-like (BTPSL) genes. These include seven *BTPSL* genes from *Metarhizium* (intronless) and two *BTPSL* genes from *Ophiocordyceps* (one intron)

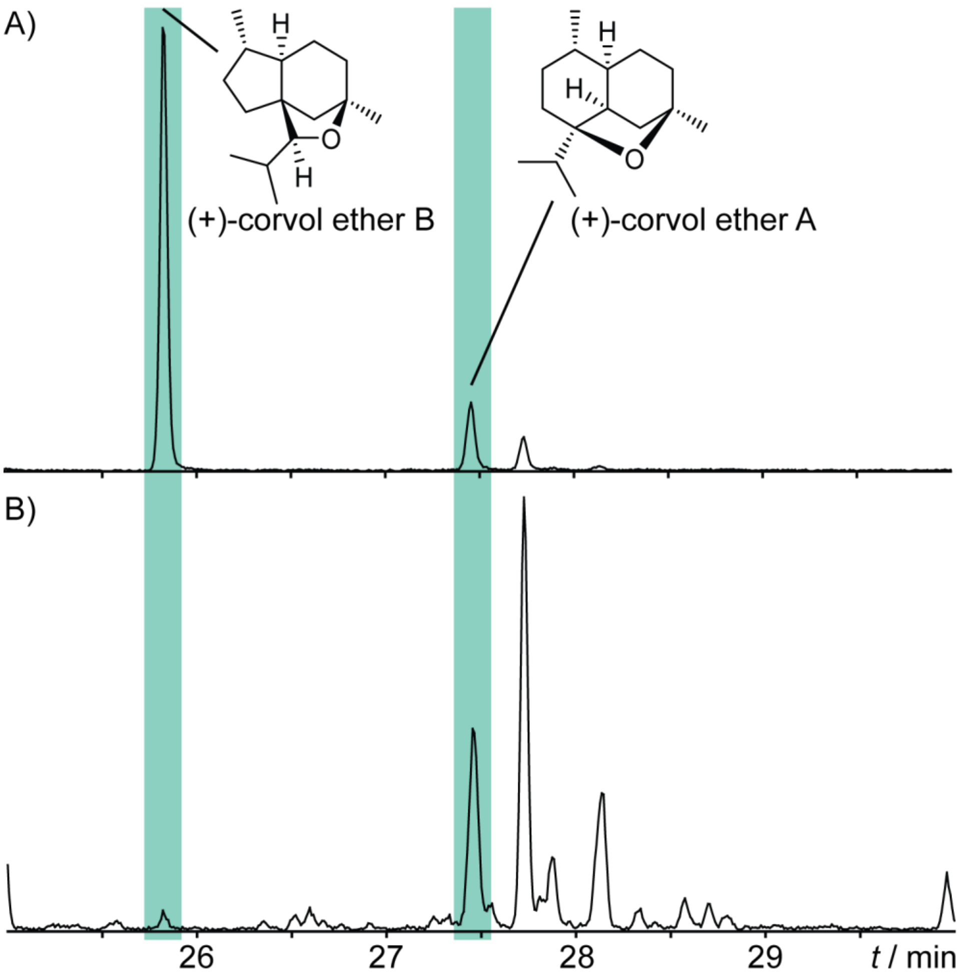

Fig S3. Terpene products identification. Total ion chromatograms of extracted incubations of farnesyl diphosphate with A) recombinant corvol ether synthase (BAJ27126) from *Kitasatospora setae* and B) recombinant MajBTPSL under the same conditions. The peaks highlighted feature the same mass spectra.

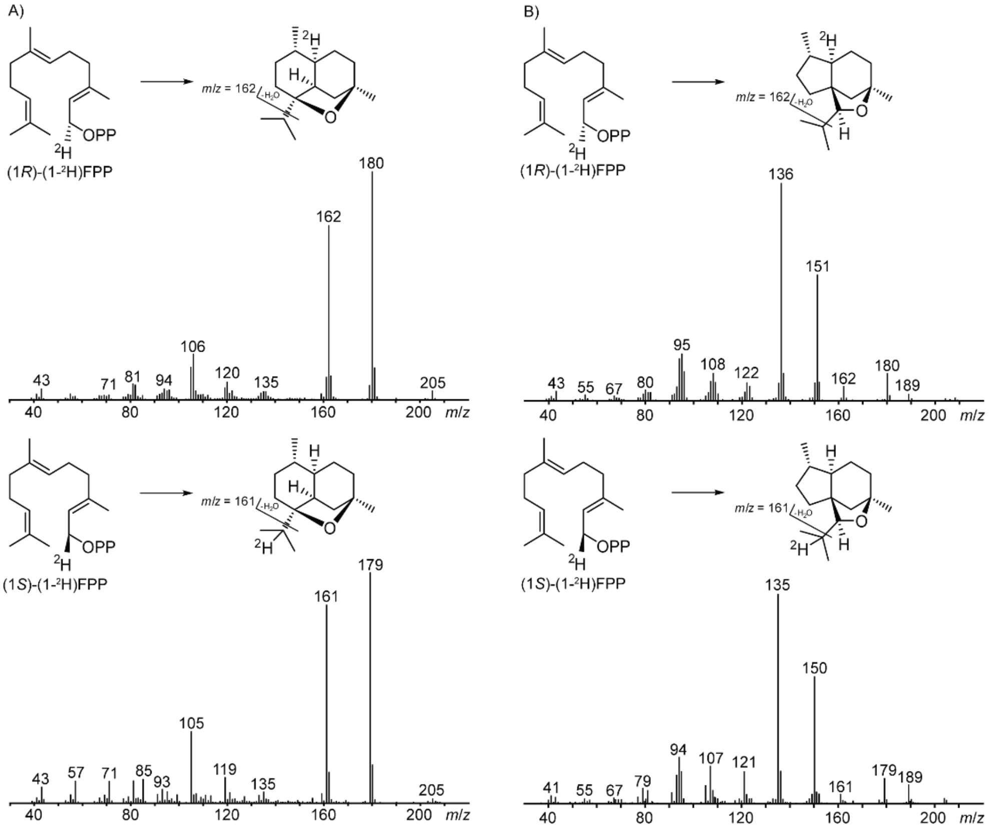

Fig S4. Mass spectra of terpenes. EI-MS spectra of deuterated A) corvol ether A and B) corvol ether B, both generated by incubating recombinant MajBTPSL and (1*R*)-(1-^{2}H)farnesyl diphosphate (top) as well as (1*S*)-(1-^{2}H)farnesyl diphosphate (bottom). The 1,3-hydride shift into the isopropyl group shows the same stereochemical course as for the bacterial corvol ether synthase from *Kitasatospora setae* indicating the same absolute configuration of the obtained products.

Table S1 BTPSL genes from eight species of entomopathogenic fungi.

Organism	*BTPSL*s	New_names	Length (aa)
Metarhizium acridum (MAC)	*MAC_05714*	*MacBTPSL*	349
Metarhizium majus (MAJ)	*MAJ_08936*	*MajBTPSL*	355
Metarhizium guizhouense (MGU)	*MGU_11447*	*MguBTPSL*	348
Metarhizium brunneum (MBR)	*MBR_10393*	*MbrBTPSL1*	332
	MBR_09977	*MbrBTPSL2*	342
Metarhizium anisopliae (MAN)	*MAN_10655*	*ManBTPSL*	355
Metarhizium robertsii (MAA)	*MAA_08668*	*MaaBTPSL*	355
Ophiocordyceps sinensis (OCS)	*OCS_03958*	*OcsBTPSL*	310
Ophiocordyceps unilateralis (XA68)	*XA68_4644*	*OunBTPSL*	342

Table S2 Primers used for gene cloning and neighbor gene confirmation

Gene name	Primers	sequence
MacBTPSL	Forward	5'-ATGGAAAAACAACACTTGAAATCTC-3'
	Reverse	5'-TCACTCAGTCGCGGACGAGGAACTTC-3'
MaaBTPSL	Forward	5'-ATGGAAAAACAAAGACTGAAAGC-3'
	Reverse	5'-CTAGACCAAGCTGCTCGTTGACTC-3'
MajBTPSL	Forward	the same as MaaBTPSL Forward
	Reverse	the same as MaaBTPSL Reverse
ManBTPSL	Forward	5'-ATGGAAAAACAAAGATTGAAAGCTC-3'
	Reverse	5'-CTAAACCAAGCTTCTCGTTGACTCAG-3'
MbrBTPSL1	Forward	5'-ATGGAAAAACAAAGATTGACAGCTC-3'
	Reverse	5'-TCAAAAGCGACGATCATCGGCATC-3'
MbrBTPSL2	Forward	5'-ATGGAAAAACAAATCTTGGAGCCTC-3'
	Reverse	5'-TTACACACTATGTCTAGAAGCAACTTG-3'
MguBTPSL	Forward	the same as MaaBTPSL Forward
	Reverse	5'-TCAGTCACAGACGAGCAACTTTTTTTTTAG-3'
BcTPS	Forward	5'-ATGACAGACGACCATCTGAAAACCGAAC-3'
	Reverse	5'-TCAGCCGCGCGCCGCCGAGTCCGCCGAC-3'
MbrBTPSL2NBF	Forward	5'-GAATGGGTCCTCTTCGTGACTTTG-3'
MbrBTPSL2NBR	Reverse	5'-CAATCATAGCCGTCAAGTTGCGCAG-3'
MAA_11029/MaaBTPSLNB	Forward	5'-ATGCCGCTGGCAGACTTGCGGTAC-3'
MaaBTPSNB1	Reverse	5'-GACTCAAGTACTGGATACGAGGTTAC-3'
MaaBTPSNB2	Forward	5'-GTCAGTCACCAGCAGATTATGCTC-3'
MaaBTPLS/MAA_08869NB	Reverse	5'-TACACAAGGCCGGCAAGAACAGTG-3'
